# Vera Peters and the curability of Hodgkin disease

**DOI:** 10.3747/co.v15i5.285

**Published:** 2008-10

**Authors:** D.H. Cowan

**Affiliations:** * Department of Medicine, Sunnybrook Health Sciences Centre and University of Toronto, and Cancer Care Ontario, Toronto, ON

**Keywords:** Vera Peters, Hodgkin disease, staging, irradiation, cure early stage

## Abstract

The middle of the 20th century hailed the realization that patients with Hodgkin disease could be cured. Through the groundbreaking work of Vera Peters, patients with a localized form of the disorder, previously thought to be incurable, were shown to be cured by extended-field radiotherapy. This important observation, although not immediately accepted, opened the minds of physicians to take more positive investigative and therapeutic approaches. Peters also introduced and championed the concept of tumour staging in Hodgkin disease and the use of prognostic factors in clinical decision-making. This novel approach led to high cure rates with radiotherapy in localized disease and provided a scientific basis for the subsequent use of chemotherapy in disseminated disease, resulting in a very high cure rate in patients with all stages of Hodgkin disease.

## 1. INTRODUCTION

### 1.1 The Initial Descriptions of Hodgkin Disease

In 1666 Marcello Malpighi, an Italian physician and scientist, was the first to describe a disease of lymph nodes and spleen that was uniformly fatal. In 1832, Thomas Hodgkin wrote about the gross pathology of seven such cases. Subsequently, in 1856 and 1865, Samuel Wilks described more cases, which included several from Hodgkin’s report. They both worked at Guy’s Hospital in London, England, although Hodgkin had retired in 1837 and it is unlikely that the two knew one another. However, Wilks recognized Hodgkin’s 1832 observations and attached the eponym Hodgkin disease to the disorder. By the early 20th century, Sternberg, Reed, and others were describing the microscopic pathology that is accepted as characteristic of Hodgkin disease 1–3.

### 1.2 Early Radiation Treatment of Hodgkin Disease

No reports of effective treatment for Hodgkin disease appeared until 1902, when Pusey reported that enlarged lymph nodes in patients with Hodgkin disease could be resolved by exposure to X rays 4. That publication was quickly followed by a Canadian report documenting similar findings in a single case later that same year 5.

René Gilbert of Geneva, Switzerland, made a major contribution in 1939 when he reported on a 17-year experience treating Hodgkin disease patients with radiation therapy. He developed a new approach that involved treating lymph node areas separately, and he administered radiation to multiple regions, not only to those involved with disease. Because of the severe skin reactions observed at high radiation doses, Hodgkin patients were generally treated to lower doses of radiation. Gilbert’s equipment was of low voltage (70–200 kV), and despite the use of low doses, he documented the disappearance of lymph nodes and mediastinal masses. Fever often resolved, and patients experienced weight gain and improvement in anemia. Gilbert calculated that survival was approximately doubled, but he emphasized that radiation treatment failed to cure the patients—relapses occurred 6.

In Toronto, Gordon Richards, a radiation treatment pioneer in North America, installed a 400-kV radiation machine at the Ontario Radiotherapy Institute at the Toronto General Hospital in 1937 7. This machine was considered superior to Gilbert’s equipment. The higher energy provided some skin sparing, which permitted the delivery of higher doses at depth within the thorax and abdomen. Richards was apparently influenced by Gilbert, because he gave added treatment to lymphatic regions with no obvious disease that were adjacent to the enlarged and involved lymph nodes.

## 2. MILDRED VERA PETERS AND HODGKIN DISEASE

### 2.1 Vera’s Early Days a

Mildred Vera Peters (always known as Vera) was born in 1911 and brought up on a farm. She received her elementary education in a one-room school house. After high school, she enrolled in the Faculty of Medicine, University of Toronto, and graduated with her md in 1934. During medical school days, she met her future husband. Their subsequent marriage was highly successful. Vera demonstrated how to balance an extremely productive career with a happy and successful family life. She became a role model for young women entering the medical profession.

Vera interned for one year at St. John’s Hospital, a women’s surgical hospital, in Toronto, where she saw a large number of patients with malignant disease. In 1935, she joined Dr. Gordon Richards at the Radio-therapy Institute in the Toronto General Hospital. She had heard lectures by Richards during medical school, and she also came to know him when he treated her mother for breast cancer. No formal training program in radiotherapy existed at the time; Peters worked as an apprentice, with Richards as her mentor 8. In 1937, the same year that the 400-kV machine was installed at the Toronto General Hospital 7, she joined the full-time staff as a junior assistant radiotherapist 9. Peters was one of the first women to have a clinical appointment in a Toronto teaching hospital. The association of Vera Peters and Gordon Richards proved to be of enormous importance to future knowledge about the management of patients with Hodgkin disease.

### 2.2 The Hodgkin Disease Story

For the first 10 years after her appointment, Peters worked with her mentor, seeing a wide range of patients and continuing to mature and hone her clinical and radiotherapy skills. She gradually became responsible for treating a large number of patients, including those with Hodgkin disease.

One day in 1947, Richards encountered Peters in the hallway at the Institute, addressed her in his usual formal manner, saying, “Dr. Peters, how would you like to review our experience with Hodgkin’s disease? All the textbooks say it is a fatal disease, but we seem to be seeing patients who are cured” 8. In later years, Peters reflected on that encounter: “That was the beginning of my career.... the more I learned about [Hodgkin disease] from charts and patients, the more I wanted to know” 8.

This was the start of two years of intensive work and, indeed, the beginning of Peters’s very productive academic career. She cared for patients during the day and brought records and patient files home at night and on weekends. Many hours were spent working at the dining room table. All the work was done by hand in this pre-computer era.

By 1949, Peters had completed the review and presented her findings to colleagues at the Toronto General Hospital. Unfortunately, Richards had died in January 1949, before Peters completed her work, and he was not aware of the outcome of the study he had suggested.

The results of radiation treatment in 113 histologically proven cases of Hodgkin disease (all reviewed by an expert pathologist) appeared in Peters’s 1950 landmark publication 10. Three extremely important messages appeared in that report:

- **The importance of a staging system.** By grouping patients according to their ultimate survival, Peters developed the first clinical classification (staging system) for patients with Hodgkin disease. The extent of disease on presentation appeared to be the most striking common factor influencing survival. Peters also noted the negative prognostic consequence of constitutional symptoms (including fever and weight loss). Table I shows the original Peters staging system. Hers was a three-stage classification, depending on the extent of demonstrable disease. The current four-stage classification b is based on that three-stage foundation. This contribution by Peters provided a method for comparing various patient series and treatment results. It also provided the first rational basis for planning treatment.
- **Prolongation of survival.** Patients at the Toronto General Hospital experienced superior survival as compared with patients previously reported. For the whole group of patients, 5-year survival doubled, and 10-year survival more than tripled. Peters showed that the survival curve flattened at about the 8- to 9-year mark. The median survival for stage i patients was more than 11.6 years; for stage ii patients, it was more than 7.8 years; and for stage iii patients, it was 1.9 years. Patients with stage i disease had a 5-year survival of 88% and a 10-year survival of 79%. These data were sufficient to suggest the possibility of a cure in patients with stage i disease. Although cure was implied, Peters did not make this claim in her first paper. Rather, she emphasized a more optimistic attitude toward prognosis in early cases if properly treated.
- **Radiation treatment.** Intensive irradiation of involved lymph nodes combined with precautionary treatment of the nodes adjacent to the involved area was required to bring about improved survival rates in patients with stage i disease.

An interested observer was Dr. Lillian Fuller. She became a renowned radiation oncologist at the M.D. Anderson Hospital in Houston, Texas, and an expert in the radiation treatment of Hodgkin disease. She had started her training in therapeutic radiology in the early 1950s in Rochester, New York. Fuller was aware of the 1950 paper, and she travelled to Toronto to meet and talk with Peters. In later years, Fuller commented that “Peters’ paper set the world on fire. Although others—for example, Gilbert—had probably cured some cases, it was Peters who demonstrated to the world that Hodgkin’s disease could be cured. No one could relate cure to stage and treatment as Vera Peters did” 11.

Nevertheless, the general response to the 1950 report was lukewarm. As Peters herself put it, “I could demonstrate [the potential for cure] on a statistical basis from the survival curves. As soon as you demonstrate something that is not part of a common belief, you run up against a lot of disbelief.... It was about ten years before it caught on—before they started to believe” 8. In the late 1950s, a Toronto physician doing postgraduate training in London, England, was greeted with a chuckle, a nudge, and the remark “Oh, you are from that place where it is believed Hodgkin’s disease can be cured!” 12

In 1956, Peters presented an update of her work at a conference in Mexico City. That update, which confirmed and built on her previous publication, was published two years later 13. The series now included 291 cases of Hodgkin disease. Cure seemed even more likely. The article stated that “a ten-year survival without reoccurrence after the initial control is necessary before one can be reasonably confident of a cure.”

The concept of cure was still not widely accepted; it remained for others to confirm Peters’s work. Important among these researchers were Easson and Russell of the Holt Radium Institute in Manchester, England. In 1963, Easson and Russell published a paper in which they made the case for the curability of Hodgkin disease 14, finally garnering acceptance by practitioners working in the field. However, widespread dissemination of information on curability took somewhat longer. A survey of the eight editions, from 1950 to 1977, of a popular and highly respected general medical textbook, *Harrison’s Principles of Internal Medicine,* revealed no mention of the curability of Hodgkin disease until the 1970s. The 1974 edition makes this statement: “The goal of therapy in Hodgkin’s disease should be to cure.... [T]he hope that Hodgkin’s can be cured was spurred by the experience of Peters” 15.

### 2.3 Further Progress by Peters and Others

After Peters made her major contribution to the control of Hodgkin disease, she began spending an increasing amount of time on her other major interest, breast cancer. But she maintained an intense interest in Hodgkin disease and published on the subject throughout her career. Of special interest were her observations about patterns of disease. She had observed these patterns in a large group of patients, first at the Ontario Radiotherapy Institute at the Toronto General Hospital, and later when patients and staff transferred to the new Princess Margaret Hospital in 1958. She had the great advantage of working in a large referral centre from which she could follow many patients in a longitudinal manner for many years. The pattern of lymph node involvement frequently allowed Peters to predict the results of investigations, including exploratory laparotomy, and to predict prognosis on the basis of an initial examination of a patient. To many, her predictions appeared intuitive, but of course they were based on the many observations she had made in numerous patients. For example, Peters observed that a patient presenting with a cluster of small lymph nodes measuring less than a centimetre in diameter in the peripheral regions of the upper torso predicted widespread disease. At exploratory surgery of the abdomen in such patients, disease would frequently be found even in small mesenteric lymph nodes. The disease in these patients was difficult to control, particularly with radiation treatment. In contrast, patients presenting with a limited number of discrete larger nodes in peripheral regions were more likely to be controlled by radiation alone and had a much better prognosis than did the former group 16,17.

Other examples of her wide interest in Hodgkin disease are reflected in important papers by Peters on the treatment of non-involved lymph node areas adjacent to the site of known disease 18 and the contribution of lymphangiography 17 to staging and the detection of deep-seated lymph nodes. Through the years, Peters continued to give many talks nationally and internationally on the subject of Hodgkin disease, and even into her retirement years, she was a sought-after consultant.

Once the curability of Hodgkin disease was established, it remained for others to carry the torch further. Henry Kaplan and his colleagues at the Stanford University Medical School played a most important role in consolidating advances in radiotherapy and the general approach to Hodgkin disease 19. At the same time, DeVita and colleagues at the National Cancer Institute in Bethesda, Maryland, demonstrated that combination chemotherapy could cure patients with advanced Hodgkin disease that was not curable with radiation alone 20. Further progress has allowed for approximately 80% of patients with all stages of Hodgkin disease to achieve cure 21.

For her outstanding contributions to the control of Hodgkin disease and her work with breast cancer patients, Vera Peters received a host of honours and awards. These included lectureships, honorary degrees, medals from prestigious oncology organizations in the United States and Europe, and the highest honour that can be bestowed upon a Canadian—the Order of Canada. Peters’s retirement included valued and enjoyable time with friends, family, and especially her grandchildren. Peters died of metastatic cancer in 1993 in the Princess Margaret Hospital, the institution she served so well for so many years.

## 3. DISCUSSION

What role did Peters take in this evolution of a cure for patients with Hodgkin disease?

There is no doubt that her work has been acclaimed internationally, both in North America and in Europe. In a chapter on Hodgkin disease in *Principles and Practice of Oncology,* Vincent P. DeVita Jr., Peter M. Mauch, and Nancy Lee Harris say that “in her landmark paper published in 1950 in the *American Journal of Roentgenology,* Peters provided evidence that patients with limited Hodgkin’s disease could be cured with aggressive radiation therapy” 22.

Just after Peters’s retirement in 1976, Henry Kaplan of Stanford University, at times a competitor, acknowledged that “twenty-five years ago, Dr. Peters introduced the first meaningful method of classifying the stages of the disease and by 1960 was getting the best results in the world with treatment by radiation” 23.

Sir David Smithers, Head of the Department of Radiotherapy at the Royal Marsden Hospital and Institute of Cancer Research in London, England, in his review of the changes in the management of Hodgkin disease over his 40-year career credited René Gilbert for beginning the work in the 1920s and 1930s, Vera Peters for the work published in 1950 and later updated, and Henry Kaplan and his group for consolidating these advances. With regard to Peters, Smithers said, “Building on the work Richards started, Dr. Vera Peters proceeded with system and sense to develop a planned radiotherapeutic attack on this disease process. This quiet, determined, and charming lady has written and spoken on the subject of Hodgkin’s disease with clarity and persistence for a number of years. She showed us the importance to prognosis both of the anatomic extent of disease and its constitutional symptoms; she established a clinical staging system, compared the results of local treatment and treatment extended to adjacent lymph node regions apparently uninvolved.... Through her, the approach of the medical profession to the disease was changed in two important respects: the staging system secured a better comparison of the results of treatment, and a more hopeful outlook was encouraged amongst her contemporaries” 19. In the dedication of his 1973 book on the disease, Smithers added, “Three people have done more to improve the results of treatment for patients with Hodgkin’s disease than any others.... this book is dedicated to Vera Peters, Henry Kaplan and to the memory of René Gilbert” 24.

It remains necessary to discuss one criticism that has been put forward by a few people. The criticism is that the patients were treated by Richards; Peters was an assistant and was merely given the opportunity to study the patients. The implication was that she has received too much of the credit. It is true that the early patients particularly were treated entirely by Richards, using a technique based on Gilbert’s work. However, when Richards introduced Peters to the question of curability, she immediately saw the importance of studying the problem and analyzing the data, and she did so! She developed the first staging system, and she demonstrated that early-stage Hodgkin disease could be cured with an adequately high dose of radiation. In other words, she related curability to early stage and aggressive radiation. Peters herself points out that she was building on the work of others (as do most clinical investigators). In the introduction to one of her papers addressing the evolution of the use of radiotherapy in Hodgkin disease, Peters wrote that “my interpretation of this evolution has been gleaned from the masterful writing of Gilbert and from my earlier experiences as a pupil of one of the esteemed pioneers of radiotherapy in this country, the late Dr. Gordon Richards” 18.

Once when asked whether she was a pioneer in Hodgkin disease, Peters modestly replied, “Well, I consider Dr. Richards to be the pioneer. It was his work, his optimism and because he kept good records that I was able to demonstrate this.... I learned firsthand from a very ingenious, kindly man, who I’m sure was a genius. He was the one who inspired.... Yes, he inspired me. After that I needed no further inspiration. The inspiration came from within from then on” 8.

With those words, Vera Peters acknowledged her mentor and chief, and in so doing, recorded an appropriate recognition of the past. Through her publications and her work with patients who had Hodgkin disease, she etched an indelible contribution for the future.

## 4. SUMMARY

Until the 1950s, Hodgkin disease was considered to be incurable. Vera Peters dispelled this gloomy prognosis for patients with early-stage disease. She carried out a careful review of histologically proven cases and established a three-stage classification. Further, she demonstrated that patients with stage i disease could be cured with aggressive radiation treatment.

## Figures and Tables

**TABLE I:** Peters’ original staging classifications 10

Stage I	Involvement of a single lymph node region or a single lesion elsewhere in the body
Stage II	Involvement of two or more proximal lymph node regions of either the upper or lower trunk
Stage III	Involvement of two or more lymph node regions of both upper and lower trunk

## Footnotes

a Information in this and later sections about the life of Vera Peters was obtained from the archives of the University of Toronto; the University Health Network Archives (Princess Margaret Hospital fonds); *Torontonensis* (the University of Toronto year-book) 1928 to 1934; the calendars of the Faculty of Medicine, University of Toronto, 1928 to 1934; family files and photograph albums; and interviews with Peters’s daughters (Ms. Sandra Clark, Dr. Jennifer Ingram) and other relatives, colleagues, and friends.

b The original classification by Peters was a three-stage system. The current classification has a number of refinements for each of stages i–iii and also adds stage iv: involvement of one or more extranodal tissues (for example, liver or bone marrow). A suffix indicates the absence (a) or presence (b) of systemic symptoms such as fever or weight loss.
